# Back to the Cradle of Cytotherapy: Integrating a Century of Clinical Research and Biotechnology-Based Manufacturing for Modern Tissue-Specific Cellular Treatments in Switzerland

**DOI:** 10.3390/bioengineering8120221

**Published:** 2021-12-17

**Authors:** Alexis Laurent, Philippe Abdel-Sayed, Corinne Scaletta, Philippe Laurent, Elénie Laurent, Murielle Michetti, Anthony de Buys Roessingh, Wassim Raffoul, Nathalie Hirt-Burri, Lee Ann Applegate

**Affiliations:** 1 Regenerative Therapy Unit, Lausanne University Hospital, University of Lausanne, 1066 Epalinges, Switzerland; alexis.laurent@unil.ch (A.L.); philippe.abdel-sayed@chuv.ch (P.A.-S.); corinne.scaletta@chuv.ch (C.S.); murielle.michetti@chuv.ch (M.M.); nathalie.burri@chuv.ch (N.H.-B.); 2 Faculty of Biology and Medicine, University of Lausanne, 1015 Lausanne, Switzerland; wassim.raffoul@chuv.ch; 3 Applied Research Department, LAM Biotechnologies SA, 1066 Epalinges, Switzerland; 4 Manufacturing Department, TEC-PHARMA SA, 1038 Bercher, Switzerland; 5 DLL Bioengineering, Discovery Learning Program, STI School of Engineering, École Polytechnique Fédérale de Lausanne, 1015 Lausanne, Switzerland; 6 School of Pharmaceutical Sciences, University of Geneva, 1206 Geneva, Switzerland; philippe.laurent@unige.ch; 7 Institute of Pharmaceutical Sciences of Western Switzerland, University of Geneva, 1206 Geneva, Switzerland; 8 Private Practice, Pharmacie du Gros-de-Vaud SA, 1038 Bercher, Switzerland; elenie@pharmabercher.ch; 9 Children and Adolescent Surgery Service, Lausanne University Hospital, University of Lausanne, 1011 Lausanne, Switzerland; anthony.debuys-roessingh@chuv.ch; 10 Lausanne Burn Center, Lausanne University Hospital, University of Lausanne, 1011 Lausanne, Switzerland; 11 Plastic, Reconstructive and Hand Surgery Service, Lausanne University Hospital, University of Lausanne, 1011 Lausanne, Switzerland; 12 Center for Applied Biotechnology and Molecular Medicine, University of Zurich, 8057 Zurich, Switzerland; 13 Oxford OSCAR Suzhou Center, Oxford University, Suzhou 215123, China

**Keywords:** biotechnological manufacturing, cell banking, cell therapy, cellular extracts, living cell therapy, primary progenitor cells, quality requirements, regenerative medicine, therapeutic products, transplantation program

## Abstract

Empirically studied by Dr. Brown-Séquard in the late 1800s, cytotherapies were later democratized by Dr. Niehans during the twentieth century in Western Switzerland. Many local cultural landmarks around the Léman Riviera are reminiscent of the inception of such cell-based treatments. Despite the discreet extravagance of the remaining heirs of “living cell therapy” and specific enforcements by Swiss health authorities, current interest in modern and scientifically sound cell-based regenerative medicine has never been stronger. Respective progress made in bioengineering and in biotechnology have enabled the clinical implementation of modern cell-based therapeutic treatments within updated medical and regulatory frameworks. Notably, the Swiss progenitor cell transplantation program has enabled the gathering of two decades of clinical experience in Lausanne for the therapeutic management of cutaneous and musculoskeletal affections, using homologous allogeneic cell-based approaches. While striking conceptual similarities exist between the respective works of the fathers of cytotherapy and of modern highly specialized clinicians, major and important iterative updates have been implemented, centered on product quality and risk-analysis-based patient safety insurance. This perspective article highlights some historical similarities and major evolutive differences, particularly regarding product safety and quality issues, characterizing the use of cell-based therapies in Switzerland over the past century. We outline the vast therapeutic potential to be harnessed for the benefit of overall patient health and the importance of specific scientific methodological aspects.

## 1. Introduction

Multiple historical and demographic factors have contributed to constitute a singular ecosystem for the development of medical technologies and related philosophies in Switzerland. Despite being referenced as an original epicenter of modern medicine and pharmaceutical innovation, Switzerland fosters a fascinating secular diversity and coexistence of therapeutic currents, which is only mirrored by the harmonious coexistence of multiple linguistic and social values within the Swiss Confederation. Specifically, in parallel to the sustained international influence of large pharmaceutical industries and the constant innovative output of the dynamic and vibrant Health Valley, this small European country harbors a unique wealth of alternative therapeutic practice diversity. Such parallel therapeutic approaches span across a wide spectrum, from homeopathy and anthroposophy, to equine immunobiologics and placental isotherapy, to magnetizers and “secret”-bearers [1,2,3]. Notably, many of these approaches are conveyed and are perpetuated through well-defined schools of thought or philosophical currents, which generally embody a holistic and patient-centered approach of health maintenance and medicine practice.

Within this highly complementary and nutritive environment, which has attracted and retained many scientific and medical minds over time, a specific therapeutic area was explored and locally democratized in Switzerland during the 20th century, namely the practice of cytotherapy (i.e., therapeutic use of cells) [4,5,6]. Originally highly experimental in nature, this therapeutic approach, based on living animal cellular active pharmaceutical ingredients (APIs), has undergone drastic evolution under multifaceted fates. Once renowned for exclusive private clinic treatments and the magistral preparation of cell-based therapeutic products, Switzerland has been following modern regulatory adaptations to better control the overall quality, safety, and clinical use of specific biological products [4]. Therefore, the official therapeutic use of living cells in particular has been technically restricted in the past two decades to Swiss national university centers and public hospitals, with some private stakeholders notably focusing on specific blood-derived or stem-cell-based applications [7,8]. Notable current clinical applications of therapeutic cell-based preparations in Switzerland therefore comprise platelet-rich plasma (i.e., for burns, Centre Hospitalier Universitaire Vaudois(CHUV), Lausanne, and others), chimeric antigen receptor (CAR-T) cells or autologous tumor-infiltrating lymphocytes enriched for tumor antigen specificity (i.e., for solid tumors in oncology, CHUV, Lausanne), autologous chondrocytes for osteoarthritis of the knee (Hôpitaux Universitaires de Genève (HUG), Geneva, and others), or pancreatic Langerhans islet transplantation (i.e., for diabetes, HUG, Geneva). Therefore, while almost all modern research centers may currently work on regenerative medicine applications and cell-based treatments, few in Switzerland may set forth extensive clinical experience in autologous cytotherapy-based approaches, and even fewer with regard to allogeneic cell-based treatments [9,10,11].

While several cell therapy protocols are currently being investigated within Swiss clinical trials, notably in the domain of cartilage regeneration, one of the precursors of standardized transplant product (TrSt) approaches has been the cultured epithelial autograft (CEA), introduced in the Lausanne Burn Center in the 1980s and remaining in specialized clinical use to this day [10,12,13,14,15,16]. In this context of highly specialized medicine provision for burn patient care, novel applications of allogeneic homologous cytotherapy products have been developed and clinically implemented in the past two decades under the Swiss progenitor cell transplantation program [9,17,18]. Therein, highly encouraging clinical results in pediatric burn patient care and promotion of geriatric refractory lower-limb cutaneous ulcer healing have been notably reported in *The Lancet* and in the *Experimental Gerontology* journals, respectively [9,19,20,21,22,23]. Importantly, said pioneer clinical research has been iteratively adapted to the successive legal updates and shifts in regulatory and medical practice-related frameworks, to remain at the forefront of continued high-quality therapeutic care provision in Switzerland [15,24]. Overall, while some striking conceptual similarities exist between the respective works of the fathers of cytotherapy and of modern highly specialized clinicians, major and important iterative updates have been implemented locally, centered mainly on product quality and risk-analysis-based patient safety insurance [11,16,18,24,25,26]. The purpose of this perspective article is to highlight some historical similarities and the major evolutive differences characterizing the clinical use of cell-based therapies in Switzerland over the past century, outlining the vast therapeutic potential to be harnessed for the benefit of overall patient health [18,26].

## 2. Genesis of Opotherapies and of Modern Cytotherapy: Drs. C.-E. Brown-Séquard and P. Niehans

Early experimental and descriptive work in the field of cytotherapy has been attributed to Dr. Charles-Édouard Brown-Séquard (1817–1894), an international and polyvalent French physician remembered for sometimes controversial yet paramount contributions to the medical fields of neurology and endocrinology [4,5,6]. At an advanced age, his work focused on the search for novel therapeutic-based means of human rejuvenation, where his reported practices may be best described by biological-based alchemy [5,6]. Therein, specific focus was allocated to the various administration modalities and holistic physiological effects of animal (i.e., dog, sheep, rabbit, guinea pig) gonad-based aqueous extracts, subcutaneously self-administered by Dr. Brown-Séquard for putative restoration of dynamism or of masculine vitality [6,27]. Self-reports of the unique claimed therapeutic results of such injections were soon published, subjectively describing effective reversal of the effects of biological age [4,6]. This approach was soon imitated by several practitioners and industries in an unregulated manner, based on the identified considerable business potential of the described injectable products, yet a great deal of scientific credibility was lost by the inventor in the process [4,28]. Many doubts were notably voiced by his medical peers, yet widespread experimentation of his opotherapy or organotherapy (i.e., use of organ-specific animal cells and extracts or derivatives, currently referred to as xenotransplantation of animal cells) around the globe contributed to democratize the name and the therapeutic concept of Dr. Brown-Séquard [4,28]. Indeed, despite the subjective medical success reported for such cell-based approaches and following the publication of the results in *The Lancet* notably, high interest was elicited among medical peers (e.g., Dr. Carl Vogt in Geneva) and in the following generation of physicians, who further refined the original approach of Dr. Brown-Séquard and who oriented their work toward tissue-specific cytotherapeutic treatments [4,5,28].

Among these recognized successors of Dr. Brown-Séquard with regard to the practice of cell-based therapies, one of the most remembered was Dr. Paul Niehans (1882–1971), a Swiss surgeon who gained vast clinical experience during the first World War in the service of the Red Cross [4]. Dr. Niehans notably fathered the for-profit and widespread yet selective practice of animal embryo-based organotherapy or “living cell therapy” [29]. This specific approach was originally based on the xenotransplantation of fresh tissue-specific bovine and ovine embryonic or fetal cells for the therapeutic treatment of the corresponding organs and tissues in patients. To this end, the fresh cell-based preparations were subcutaneously or intramuscularly injected in complex suspension form, in view of obtaining specific therapeutic actions through postulated “homologous cell homing” or systemic rejuvenation effects similar to those sought and described by Dr. Brown-Séquard [4]. Therein, extensive clinical practice, experience, and relative global fame were achieved by Dr. Niehans in Montreux on the Léman Riviera, notably remembered to this day for the development of topical product forms dedicated to cutaneous revitalization, known under the La Prairie brand. Similarly to the effects procured by the widespread attention gained by Dr. Brown-Séquard in his time, many celebrities (e.g., Charlie Chaplin, Pope Pius XII) soon visited Dr. Niehans in Montreux to be treated using “living cell therapy” for various medical conditions [4]. Additionally, mirroring the detrimental effects of unregulated business approaches of Dr. Brown-Séquard’s “elixir”, major professional concerns were soon expressed and documented around the commercial practice of Dr. Niehans’s “living cell therapy”, with several medical guidelines being opposed to its use (e.g., for oncology patients) [29].

Despite documented safety concerns and the lack of sound peer-reviewed and published clinical evidence around the original form of Dr. Niehans’s cytotherapy practices, several scientific concepts have been loosely inspired or derived from them and remain in current use [4]. Furthermore and specifically, such modernized practices have further forged the continued interest of modern esthetics professionals, notably focusing on optimization of cutaneous aging sign minimization [30,31,32]. However, despite the local fame and status of the work of Dr. Niehans, which has inspired several categories of products found in Swiss pharmacies over the years, business-oriented and safety-related considerations have led local and national authorities to technically outlaw many historical practices related to original “living cell therapy” [33]. Indeed, with an exponential surge in the interest for such costly “cures” in Swiss private clinics, notably from Russian, Middle Eastern, and pan-Asian markets, the unregulated development of these specific activities was uniformly capped in the 2010s [24,33]. Therein, the freedom of medical prescription and magistral formula manufacture was replaced with safety and quality-based considerations applying to all regulated therapeutic products and biological transplants, respectively [24,34,35]. However, due to the large residual demand for practices inspired by “living cell therapy” and to jurisdictional conflicts between local and national Swiss regulating bodies, numerous remaining practices exist in a qualified grey zone [4].

## 3. Evolution and Standardization of Specific Therapeutic Preparations and of Cell Therapies in Switzerland during the 20th and 21st Centuries

In parallel to the efforts of countering senescence and the commercialization of rejuvenation solutions by Dr. Niehans, various alternative and highly specific cell-based therapeutic approaches were adopted during the 20th century in standardized clinical settings [36,37,38,39]. This was notably enabled by respective technical developments in the fields of bioengineering and biotechnology, with the evolving capacity to isolate tissues and cells in vitro for organism-independent culture and the development of biocompatible scaffolds for therapeutic grafts and artificial tissue constitution [39]. A highly specific application of cultured cell-based therapy consisted of the development in Boston in the 1970s by Green et al. of a technique for the in vitro preparation of pluri-stratified autologous keratinocyte sheets, to be therapeutically applied on burn patients [12,14]. Recognizing the vast therapeutic potential of such cell-based tissular constructs for quasi-orphan medical conditions, the Lausanne University Hospital (CHUV) immediately dispatched personnel to Boston, to learn and import such practices back to the local Romand Burn Center in 1985 [13,16].

**Figure 1:**
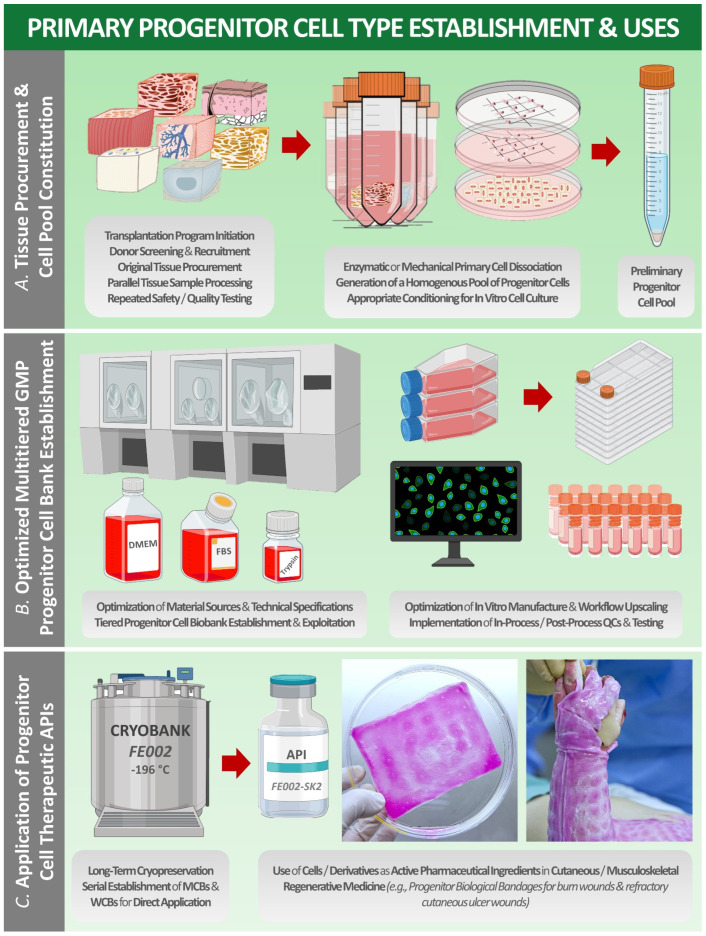
Schematic technical overview of the multiple steps performed in modern settings for the appropriate sourcing, manufacturing, and formulation of clinically compatible progenitor cell-based therapeutic products or standardized transplants. (**A**) Tissue procurement and preliminary progenitor cell pool constitution step. (**B**) Multiparametric technical optimization phase and multitiered GMP biobanking of primary progenitor cells. (**C**) Example of a clinical application of extemporaneously reconstituted skin-derived progenitor cells (e.g., FE002-SK2 fibroblasts), topically applied as viable cells seeded on a collagen scaffold (e.g., PBB product) for pediatric burn patient care in Lausanne under the Swiss progenitor cell transplantation program. API, active pharmaceutical ingredient; DMEM, Dulbecco’s modified Eagle medium; FBS, fetal bovine serum; GMP, good manufacturing practice; MCB, master cell bank; PBB, progenitor biological bandage; QC, quality control; WCB, working cell bank.

**Figure 2:**
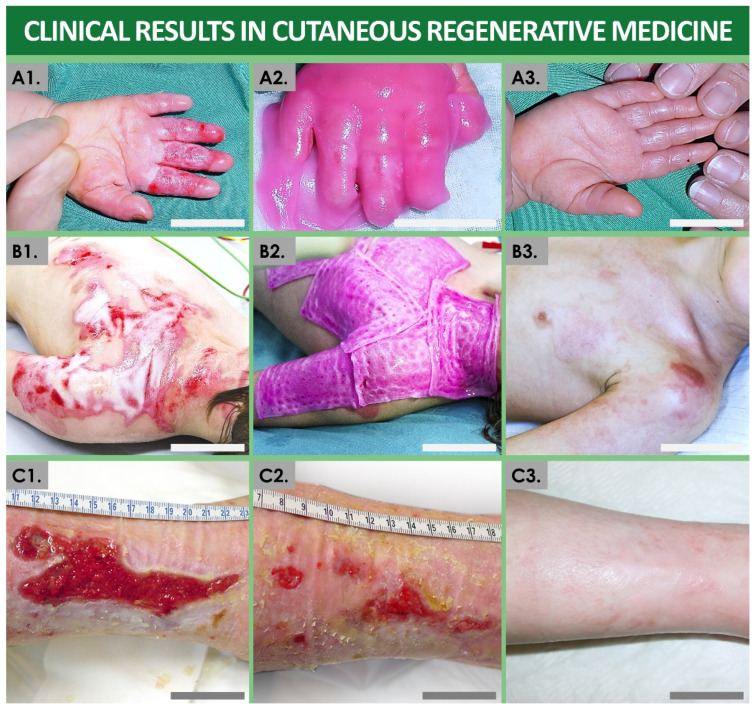
Illustrative overview of obtained clinical results using homologous skin progenitor cell-based PBBs in Swiss allogeneic cutaneous regenerative medicine. (**A**) Second-degree and third-degree pediatric hand burn wound (i.e., caused by scalding liquid). Photographic representations of the lesions after early debridement (**A1**), after PBB application (**A2**), and after six weeks of treatment (**A3**). Scale bars = 2 cm. (**B**) Second-degree deep pediatric torso burn wound (i.e., caused by scalding liquid). Photographic representations of the lesions after early debridement (**B1**), after PBB application (**B2**), and after six weeks of treatment (**B3**). Scale bars = 5 cm. (**C**) Refractory and painful post-thrombotic cutaneous ulcer lesions treated weekly using PBBs. Photographic representations of the lesions at the time of the treatment initiation (**C1**), 11 weeks later (**C2**), and 15 months later during follow-up monitoring (**C3**). Scale bars = 4 cm. PBB, progenitor biological bandage. Modified and adapted with permission from Laurent et al., 2020 [18].

**Figure 3:**
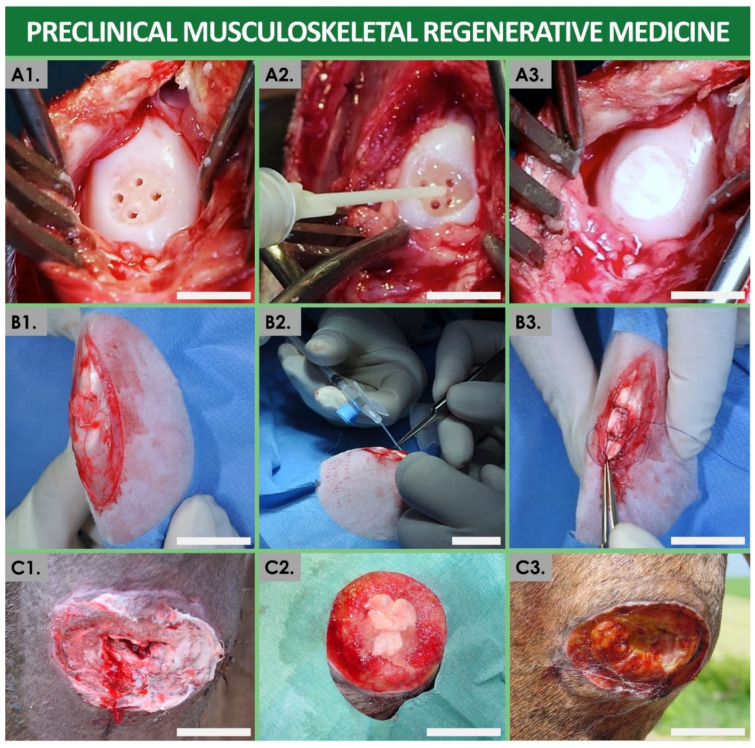
Illustrative overview of obtained preclinical results using homologous progenitor cell-based preparations in Swiss musculoskeletal regenerative medicine. (**A**) Illustration of cartilage lesion treatment using human progenitor chondrocyte-based constructs in a caprine model of full-thickness articular cartilage defect. Microfracture drill holes are created in the subchondral bone plate (**A1**), fibrin glue is locally administered in the lesion (**A2**), and the bioengineered cell-laden therapeutic construct is securely implanted in the defect (**A3**). Scale bars = 8 mm. (**B**) Illustration of tendon tissue lesion treatment using human progenitor tenocyte-based hydrogels in a lagomorph model of patellar tendon defect. The mid-thickness tendon tissue defect is evidenced (**B1**), the therapeutic cell-laden hydrogel product is injected locally into the partially sutured defect (**B2**), and the tissue defect is closed and sutured (**B3**). Scale bars = 2 cm. (**C**) Illustration of a case study of volumetric soft tissue loss in the joint of a female pony, treated with complex equine progenitor cell-based progenitor biological bandages (ePBBs). The initial wound (**C1**) was appropriately cleaned and treated with PBBs (**C2**), leading to rapid healing evolution of the wound after three days (**C3**). Scale bars = 2 cm. ePBB, equine progenitor biological bandage. Modified and adapted with permission from Laurent et al., 2020, 2021a, and 2021b [43,44,45].

Over the past 40 years, such practices have been iteratively ameliorated, and skin cell-based products have been therapeutically applied in hundreds of burn patients in Lausanne, with parallel therapy development efforts made around the Zurich Burn Centers [11,16,40]. Despite the proven life-saving aspects of such treatments and the proven therapeutic gains for patients (i.e., reimbursement by Swiss basic health insurance of CEAs), several technical bottlenecks remained, such as the lack of early wound coverage solutions due to lengthy autologous cell manufacture steps [11,15,16]. Therefore, applied research from the 1990s in the field of developmental cell biology yielded a new generation of cell-based therapeutic products for cutaneous regenerative medicine, based on the allogeneic transplantation of viable human-tissue-specific primary progenitor cells (Figure 1) [9,11,20]. Therein, the most documented clinical application has consisted of the therapeutic use of progenitor biological bandages (PBBs), applied for early wound coverage and optimal healing promotion of pediatric burns and geriatric cutaneous ulcers in particular (Figure 2) [9,19,23]. Over two decades of continued clinical experience with such PBB products (i.e., over 3000 units clinically applied in 2013–2021) have outlined the tangible therapeutic gains procured by such cell-based preparations, despite the remaining margins of improvement regarding technical aspects of product manufacturing and clinical administration [11,23]. Additionally, similar preclinical work in musculoskeletal regenerative medicine for repair of cartilage and tendon tissues using similar homologous progenitor cell therapy approaches have recently yielded encouraging results, prompting further applied research and cytotherapeutic product development efforts (Figure 3) [41,42,43,44,45].

Furthermore, based on a historical review of the high diversity of specific or outstanding treatments proposed in the Swiss market over the past century, several examples were identified as worthy of mention herein (Table 1). Indeed, such products represent important local historical landmarks, and are highly illustrative of the movement toward standardization and pharmaceuticalization of “alternative” treatments in Switzerland during the 20th century. Although the presented preparation types may not be assimilated as cytotherapies, many have revolved around the use of specific tissues, extracts, immunoglobulins, or serum-based components (Table 1).

## 4. Implementation of GMPs and Modern Regulatory Frameworks for Cell-Based Therapies in Switzerland

Major legislative and regulatory updates were implemented in Switzerland in 2007, with direct consequences for the manufacturing and clinical practices of CEA or PBB preparation for burn patients, as described previously [15,24]. Notably, stringent quality requirements regarding APIs and finished product manufacturing facilities were enforced, prompting university hospitals to massively invest to create fully GMP-compliant cell production platforms for their own use [16,24,25]. An example of this type of infrastructure is the Lausanne Cell Production Center (CPC), authorized and purposed since 2015 with the contract manufacturing of patient-specific cell-based therapies and PBBs for the Lausanne Burn Center and the CHUV Orthopedics Service [16]. Although GMP manufacture ensures enhanced levels of therapeutic product quality and safety for the patients, such processing has come to constitute a main driver of direct costs for therapy manufacture and overall care provision, as reported by numerous groups [24,52,53,54]. In addition to manufacturing-related quality requirements, regulatory aspects of cell therapy administration in university hospital settings were modified in Switzerland after 2007 [24]. Due to the discontinued possibility of using the magistral preparation pathway, as previously mentioned, the continued use of cell therapies for burn patients has been iteratively questioned, yet never interrupted in Lausanne and qualified as pertaining to compassionate use [11]. Current requirements would tend to indicate a necessity to pursue market authorization pathways and devise new clinical trials, despite the extensive available clinical experience for CEAs and PBBs [16].

From a technical viewpoint, bioengineered products such as those considered herein for the treatment of burn patients by delivery of therapeutic cells are classified as standardized transplant products (TrSt) in Switzerland and as combined advanced therapy medicinal products (ATMPs) in Europe (Table 2) [16,25,34,35].

Therein, the inherent steps of substantial manipulation of the biological materials (i.e., cell expansion during in vitro culture) for TrSt elaboration must be notably performed under GMP-accredited systems and infrastructures, which are derived from classical pharmaceutical industry guidelines [16,24]. Notably, enforcement of such requirements has been reported to progressively limit and eventually reduce the quantity of therapies or products effectively reaching clinical implementation. This aspect may be perceived as highly detrimental to university hospitals in particular, with fundamental questioning of the use of historical and clinically proven therapeutic interventions (e.g., cultured autografts for burn wounds) [16,55]. Indeed, prompted by pharaonic direct costs of GMP cell manufacture and the weight of regulatory file submissions, public and private stakeholders have developed innovative approaches to maintain the ongoing use of cell therapies. Such proceedings were deemed essential for legal and regulatory compliance, while perpetuating the provision of high-quality therapies to vulnerable patient populations [16,24].

Up until recently, hospitals were the main drivers for the development of autologous cell therapies in Switzerland. As mentioned previously, the Lausanne Burn Center has continuously been clinically applying CEAs and cultured dermal–epidermal autografts (CDEA) for the past 35 years [16]. Additionally to the in-house use of such transplants, a number of cell-based preparations have been conditionally furnished to the Zurich Burn Centers for both children and adult patients [16]. Current high interest is set on the commercial development of products containing skin cell cultures to be combined with appropriate matrices. The Zurich Burn Center has recently collaborated with an industry for such developments, where the treatment of two patients under compassionate use has been reported to date [56,57]. The technical capacity of an industry to cover global territory with volatile living autologous grafts and to deal with logistics efficiency has been under discussion recently. Specifically, there is an emerging framework and working group advocating for the importance of “point-of-care” manufacture for advanced therapies, emphasizing the advantages of cell manufacture in close proximity to hospitals and patients. Such frameworks are being implemented in countries with large geographical areas, such as Canada [58].

As concerns autologous cartilage cell transplantation, the Lausanne University Hospital has been a leader in Switzerland in the implementation of autologous chondrocyte implantation (ACI) protocols, adapted with human platelet lysates (HPL) instead of fetal bovine serum (FBS) for cell culture steps (ClinicalTrials.gov Identifier: NCT04296487). In addition, a novel and promising “nose-to-knee” approach of chondrocyte-based treatment has been recently developed in the University Hospital in Basel, and was integrated in multicentric clinical trials (ClinicalTrials.gov Identifiers: NCT01605201 and NCT02673905) [10].

Overall, tissue-specific cell therapies have historically been at the forefront of advanced patient clinical care in Switzerland. Allogeneic cell therapies have been promoted in recent years for the standardization of therapeutic products and the potential development of off-the-freezer and off-the-shelf therapies [26]. Notably, the Swiss Stem Cell Foundation has acted as a precursor for the definition of GMP structures and processes for the manufacture and storage of stem cell sources in Switzerland (https://sscf.ch, accessed on 26 November 2021). Generally, partners for clinical trials have been identified for numerous medical conditions, where the private industrial sector collaborates in view of tangible technological transposition and product development (Table 3). Specifically, all of these programs integrate in-house or close local collaborations with university hospitals for continued clinical work. Specifically, university centers or public hospitals usually do not develop finished therapeutic products and therapies to be submitted for market approval. Therefore, technologies are developed and transposed by industrial partners using private funding and infrastructures (Table 3). Due to recent irregularities in product regulatory classification and approval procedures, such interactions between public and private collaborators have been difficult, especially if pre-existing intellectual property or know-how is involved in the negotiations.

Due to the aforementioned regulatory hurdles currently hampering the effective development and transposition of cell-based therapeutic products in Switzerland, diversified approaches and pathways have been investigated. Among these approaches are hospital exemptions, compassionate use, exceptional authorizations, orphan drug pathways, magistral or officinal preparations, and the homologation of novel cell-based components in recognized repositories such as a pharmacopeia [16,59]. Continued innovation and close collaboration with regulators and policy makers therefore constitute cornerstones to ensure clinical progress and an effective translational drive toward optimized patient therapeutic care in regenerative medicine.

Specifically and as previously stated, the highest importance is set at individual institutional levels, in close collaboration with the different internal research and clinical groups, for facilitation of the dialogue with national health authorities around the implemented and novel cell therapies of interest. Notably, continued clinical management of in-house burn patients in the Lausanne Burn Center with cultivated skin cells was only made possible through iterative exchanges and discussions with the competent regulatory bodies following the multiple changes and updates in the Swiss legal frameworks over the years [24]. Therefore, it may be overall assessed that the continued institutional work and multifactorial support provided by Swiss university hospitals in particular have played a major historic role in favoring the progress of local and highly specific medical innovation in the field of cell therapies.

## 5. Safety and Quality as Paramount Attributes in the Modern Manufacture of Cell Therapy Products

Despite the potential to qualify the described recent and drastic regulatory requirement modifications as disproportionate, such were introduced and enforced following the same logic and goals pursued by national medicines agencies, namely for insurance of product safety and quality [24]. Of particular current importance, the transplantation of viable human or animal cells presents tangible inherent risks to patient health in the absence of the appropriate extensive screening and testing [26]. Critical importance is therefore set on the exclusion of the potential transmission of viruses, bacteria, and alternative extraneous contaminants to the patient by the biological-based products. Additionally, qualification of specific cell sources is necessary to exclude the potential for immune-response eliciting in the recipient or tumorigenicity [26]. When considering allogeneic progenitor or perinatal cell transplantation, several positive aspects related to safety and quality have been reported, documenting the adequacy of such biological materials for therapeutic product or standardized transplant preparation [18,60,61,62,63,64,65].

Specifically, selected cultured primary progenitor cells were shown to meet the stringent technical requirements for the development of allogeneic biological-based therapeutic products or standardized transplants [18,26]. Indeed, cultured primary progenitor cells have benefitted from extensive industrial applications since the 1970s, notably in the field of vaccine product testing and manufacturing activities (e.g., WI-38 and MRC-5 cell types), demonstrating high stability and adequation for acting as industry standards [66,67,68,69]. Following adequate bioprocessing from fetal tissues, such primary cells may be characterized by pre-terminally differentiated phenotypes, considerable expansion and regeneration promotion potential, and low immunogenicity or tumorigenicity [17,20,69]. Additionally, such cell types do not require the presence of feeder layers or the use of defined growth factor cocktails for in vitro expansion, in contrast to primary keratinocytes or stem cells for example. These relatively simple technical requirements notably lead to reduced overall direct costs of cell manufacture [26]. Tissue-specific and stable phenotypes or functionalities of primary progenitor cells, obtained after appropriate tissue-specific bioprocessing, enable the manufacture of homogenous cell populations that may eventually be applied in homologous therapeutic approaches [18].

As previously stated, the robustness of selected primary progenitor cell sources is mainly based on a conservative cell isolation process, extensive expansion capacities, minimal growth requirements, excellent biocompatibility with engineered scaffolds, and high resistance to oxidative stress [18]. Multitiered primary progenitor cell biobanks may be rapidly and efficiently established and qualified under GMP standards within optimized manufacturing workflows [17,70]. Notwithstanding the restriction of use of cellular materials in the last third of the qualified in vitro cell type lifespan, specific models have established the technical potential for the preparation of several billion cryopreserved therapeutic cell doses following a single original organ donation [26]. Such possibilities have paved the way toward standardization of biological-material-based therapeutic products and cell therapies, in a similar way to industrial biotechnological substrates, as mentioned previously [69]. Of critical importance for selected clinical applications (i.e., extensive burn wounds), such manufacturing approaches enable the on-demand availability of standardized and consistent cryopreserved materials. Therein, the delays in clinical treatment of burn patients, associated with high costs and contamination risks, may be drastically reduced [16,23].

## 6. The Swiss Progenitor Cell Transplantation Program and Two Decades of Clinical Cytotherapy Experience in Lausanne

Applied developmental cell biology studies undertaken in the 1990s in Lausanne have constituted the basis for allogeneic therapeutic applications of human primary progenitor cells [17]. It was outlined and reported that such cell types, adequately isolated from donated perinatal tissue samples, presented considerable therapeutic potential. Therefore, a critical systematic approach was adopted for further developmental work eventually resulting in clinical applications [18,71]. Specifically, several key historical concepts were adapted, such as the homologous use of tissue-specific cells for therapeutic purposes (e.g., experimental works reported by Drs. Brown-Séquard and Niehans), the establishment and tiered banking of primary progenitor cell sources (i.e., as laid down by Dr. Hayflick in the 1960s around the WI-38 cell type and others, as reported in prestigious journals such as *The Lancet* and *Experimental Gerontology*), and applicable legal frameworks in Switzerland (i.e., laws on therapeutic products and on transplantation) [4,18,34,35,66,68,72,73,74,75]. The integration of such concepts and holistic considerations of aging and cellular repair in a therapeutically oriented and harmonized methodological framework laid the foundations of the Swiss progenitor cell transplantation program [18]. In detail, the therapeutic use of human primary progenitor cells has been devised within a transplantation program since 1991, and was registered with the Swiss Federal Office of Public Health and later with Swissmedic, the Swiss therapeutic products agency [18].

Specifically, appropriate consideration of donor consent and anonymity insurance were built as cornerstones of the ad hoc transplantation program, with extremely well-defined rights and obligations with regard to ownership of established cells banks and related technical know-how [17,18].

This was specifically inspired by historical events related to the procurement of the original tissues serving for the establishment of the WI-38 or HeLa cell sources in particular [76]. Furthermore, technical aspects of the transplantation program were adapted to fit the stringent material processing conditions and exhaustive traceability prerequisites of clinical-grade cell banking [17]. Therein, use of a transplantation program platform was identified as technically optimal, with the appropriate and compartmentalized multidisciplinary collaboration necessary to obtain a preliminary progenitor cell lot (Figure 1). Following approved workflows and technical specifications relative to the donated materials after voluntary pregnancy interruptions, the constitution of tissue-specific primary progenitor parental cell banks (PCBs) served as the first step of therapeutic product development [26]. Thereafter, it was shown that when appropriately derived and maintained, such cell sources may furnish sufficient progeny materials for decades of development in translational regenerative medicine [18].

As previously mentioned, the most clinical experience gathered over the past 20 years in Lausanne has revolved around the therapeutic use of skin-derived primary progenitor fibroblasts for managing burn wounds and refractory cutaneous ulcers (Figure 2) [9,21,23]. The technical simplicity, bioprocessing robustness, and requirements for relatively small cellular API therapeutic doses have enabled continuous and efficient manufacture of clinical progenitor fibroblast lots under GMP standards [26].

Extemporaneous therapeutic product preparation by direct off-the-freezer seeding of viable dermal progenitor fibroblasts (e.g., FE002-SK2 cell source) on equine collagen scaffolds was used to constitute the progenitor biological bandage wound coverage solution (Figure 2) [23]. PBBs could be iteratively clinically applied during bandage exchange procedures, without the need for stappling. In reported clinical cases, skin reconstruction was rapidly promoted by the therapeutic use of PBBs, which allowed for restoration of high tissue elastic properties and pigmentation balance. Additionally, reduction in pain, scar hypertrophy, retraction, tissue inflammation, or the absence of the necessity for additional skin grafting were documented [9,19,21,23]. From a technical point of view, adequacy of the methodology of the Swiss progenitor cell transplantation program was confirmed with the approval of use of the FE002-SK2 skin-derived cell source in clinical trials (i.e., authorized by the FDA, TFDA, and PMDA) [26]. Over two decades of clinical use and product application in multicentric clinical studies have contributed to outline both the safety and the beneficial therapeutic effects of cell sources used in PBB constructs, in phase I and II clinical trials in Switzerland or in Asia (e.g., ClinicalTrials.gov Identifiers: NCT02737748 and NCT03624023) [26]. Diversification of the clinical indications and delivery methods for these progenitor cells has produced excellent results for the treatment of persistent cutaneous ulcers, autograft donor site wounds, or chronic cutaneous affections such as eczema [18,19]. Building on these encouraging results in cutaneous regenerative medicine, parallel and current efforts are devoted to similar yet specific approaches of homologous progenitor cell therapeutic applications in musculoskeletal regenerative medicine [77,78,79,80]. In particular, recent advancements in the field of cartilage and tendon tissue reconstruction have also yielded encouraging safety results, prompting the further establishment of a therapeutic rationale of a tissue-specific, progenitor-cell-based approach in highly specialized medicine [44,45].

## 7. Original Tissue-Specific Cytotherapeutic Concepts Enhanced by Biotechnological Manufacturing Processes and Modern Bioengineering Solutions

The conclusions of Dr. Brown-Séquard pertaining to the tissue-specific secretory functions and the homologous therapeutic potential of biological extracts led him to experiment with the effects of orchitic extracts on himself for postulated restoration of vitality [5,6]. Similarly, the use of tissue-specific preparations was adopted in various therapeutic approaches, among which are the “living cell therapy” of Dr. Niehans, the use of tissue-specific equine immunoglobulins (e.g., serocytotherapy), or the use of specific starting materials in classical homeopathy [2,3,4,81]. Therein, the common rationale has been to work tissue by tissue or organ by organ, for optimal focus on the deployed therapeutic effects of the considered APIs or specific derivatives. In resonance with such concepts, a homologous therapeutic approach was adopted for the Swiss progenitor cell transplantation program [18]. Due to the technical possibility and relative ease of establishing individual tissue-specific mammalian cell types and to derive the related progeny materials in standardized biobanking workflows, homologous approaches were investigated in Swiss progenitor cell regenerative medicine (Table 4) [18,43].

Overall, the optimal consistency and high stability of the selected progenitor cell sources, simultaneously derived from one organ donation, have presented vast potential for tangible cellular product development under the strictest safety- and quality-driven requirements of manufacturing (Table 4) [18]. Despite the finite in vitro lifespan of primary progenitor cells, minimal processing requirements suffice for sustainable GMP manufacturing at industrial scales, as attested by the widespread use of the WI-38 or MRC-5 cells [18,68]. Following optimized and standardized multitiered cell banking models, the efficient establishment, transposition, and eventual utilization of high therapeutic value biological material sources is enabled. Furthermore, localized homologous applications of therapeutic progenitor cells enable high sustainability of manufacturing, as individual API doses (e.g., 0.5 × 10^6^ to 3 × 10^6^ cells/product unit for skin, cartilage, and tendons) are relatively smaller than cellular doses classically administered in stem cell-based therapies (e.g., 10^8^ to 10^9^ cells/dose) [18,82]. Therefore, and as stated, the sparing use of cryopreserved materials may lead to the production of several billion finished products for each progenitor cell source [26]. Of high current importance, it may be assessed that maximized safety, quality, and efficiency of optimized industrial manufacturing schemes for progenitor cellular APIs cost-enable the development of innovative therapeutic products and ensure the on-demand clinical availability of end-products.

Of note, the current state of the art in Swiss allogeneic progenitor-cell-based regenerative medicine is summarily presented and referenced, for a selection of considered primary cell types, in Table 4. Therein, and in addition to the previously described applications of progenitor skin cells within progenitor biological bandages in cutaneous wound care, alternative tissue-specific applications of dedicated progenitor cell sources may be distinguished or differentially described based on the potential homologous therapeutic indications, and on the various and specific bioengineering product formulation parameters [18]. Indeed, the optimal choice of appropriate scaffolds and cell delivery vehicles is critical for the insurance of finished product safety and quality. Tissue engineering applications requiring volumetric supplementation and biomechanical functions of the grafted construct (e.g., bone, cartilage, or intervertebral disc grafts) favor the use of porous polymeric scaffolds, to be appropriately seeded with cellular components and mechanically or chemically conditioned before implantation [41,44,78,79,80]. Alternatively, in case of topical or subcritical wounds and defects, formulations based on moldable (e.g., collagen sheets) or injectable (e.g., hyaluronic acid gels) vehicles are currently considered in Switzerland for skin, tendon, or muscle affections [23,45,77,82]. Ultimately, the dynamic biological parameters of the various considered combination products are necessarily taken into account to respect the founding triad of tissue engineering (i.e., cells, scaffolds, and bioactive molecules).

Future perspectives and considerations for potential novel cell-based therapeutic product development in Switzerland may be notably assessed from technical or regulatory viewpoints. As regards future developments of cellular APIs, efforts and resources shall, in all probability, be allocated toward the identification of additional optimal therapeutic cell sources, modification of such cell sources using emerging molecular and genetic engineering tools (e.g., CRISPR/Cas9), or processing of the selected cells and their byproducts into derivatives (e.g., exosomes, secretomes). From a regulatory point of view, investigation of new or existing pathways and strategies of technology and therapy development will necessarily drive the effective translation and transposition of products and processes yielded by biotechnological innovation. Therefore, and given the considerable economic constraints existing for industries developing cell-based therapies, university hospital centers appear as catalysts of future preclinical and clinical work in specialized regenerative medicine, as demonstrated by the recent progression and current state of cell therapy development in Switzerland [18]. Importantly, such ecosystems should be maintained, fostered, and specifically developed to ensure that current and future therapeutic needs of patients are consistently and continuously met.

## 8. A Forward Return Back to Tissue-Specific Cell-Based Therapeutic Extracts for Individualized Regenerative Medicine

Despite widespread efforts to achieve successful transplantation of living cells in many cell therapy approaches, poor viability of transplants has always constituted a major technical bottleneck. However, while specific applications require conserved cell viability for the maintenance of functionality, new evidence suggests that cell-based and cell-free products bear significant therapeutic potential [84,85,86,87,88,89]. Therein, much scientific and industrial focus has been set on cellular fractions such as lysates or exosomes, for eliciting of repair or regeneration effects comparable to those of living cells [84,87,88]. Such approaches have presented several advantages, among which are the alleviating of specific safety concerns and facilitated logistical workflows. Overall and as previously mentioned, several technical reasons prompt the further development of progenitor cell derivatives or complex cell extracts for appropriate inclusion in therapeutic products or medical devices [70].

A main difference that should be underlined between the original concepts of “living cell therapy” and modern regenerative medicine practices consists of the mode of administration or related pharmacokinetic considerations around the formulated cell-based materials. While the primitive forms of cytotherapeutic products were injected subcutaneously or intramuscularly and were supposed to autonomously distribute by tissue-specific homing, modern approaches prefer a homologous local administration regimen, based on the principles of tissue engineering [5,18]. Furthermore, modern cell therapies require extensive infrastructure and exhaustive regulatory documentation. Therefore, the previously mentioned and precursor Swiss autologous transplantation programs for skin and cartilage cells have laid the foundations for interdisciplinary teams to assure optimized manufacture in relation to infrastructure and delivery of therapeutic products to clinician teams. These setups will then eventually be able to accommodate allogeneic cell therapy practices, once they reach sufficient maturity levels.

Overall, parallels may be drawn between the original work of Dr. Brown-Séquard, who chose to use aqueous or macerated glycerin extracts for the preparation of his tissue-specific products, and the current work of highly specialized clinicians in Switzerland [18,27]. While technical means of cell-based API processing and quality controls have drastically evolved, conserved aspects of the original rationales comprise specific tissue processing in view of homologous uses, and the derivation of biological material-based extracts, formulated into stable yet effective product forms [9,43,44,45,70]. Therefore, the general technical goal pursued by Dr. Brown-Séquard and by modern research groups consists of the eventual obtention of stable tissue-specific cell-based preparations, to be therapeutically used alone or in balanced combinations depending on the clinical case at hand. Such successful proceedings would constitute a truly individualized and holistic approach to the restoration of structural and functional parameters of treated patients.

## 9. Conclusions

Integrating local historical elements of cell-based therapeutic rationale, technical manufacturing optimization, and modern specialized medical practices assuring utmost patient safety, innovative and potentially holistic approaches of regenerative medicine are being pursued in Western Switzerland. Without documentation and knowledge transmission by local Swiss pharmacists, many of the historic aspects of primitive cell therapies and alternative therapeutic products would have disappeared. Striking parallels (and major differences) may be observed, based on geographical proximity and alignment of technical or therapeutic goals, between original applications of cell therapy in Montreux and current treatments administered at the Lausanne University Hospital. Notably, the Swiss progenitor cell transplantation program has enabled the gathering of two decades of scientifically sound clinical experience for the management of cutaneous and musculoskeletal injuries and affections, using homologous allogeneic cell-based approaches. While some conceptual similarities exist between the respective works of the fathers of cytotherapy and of modern highly specialized Swiss clinicians, major and important iterative updates have been implemented, centered on product quality and risk-analysis-based patient safety insurance. Overall, it may be stated that vast therapeutic potential remains to be harnessed around the use of progenitor-cell-based preparations in particular, for the tangible benefit of patient health.

## Figures and Tables

**Table 1:** Selective overview of various therapeutic preparation types historically linked to the Swiss market. Although not considered as cell therapies, the listed preparation types all revolved around the use of specific tissues, biological extracts, immunoglobulins, or serum-based components. Therein, product specificity constituted a cornerstone of most therapeutic principles, despite technological or formulation differences. APIs, active pharmaceutical ingredients; SPE, sheep placental extract.

Preparation Type/Name	Technical Description	Therapeutic Rationale, Examples, and Known Swiss Manufacturers
Sheep placental extracts (SPEs)	Processed ovine placenta (by hydrolysis or mechanical separation) for obtention of complex protein extract solutions.	Use of ovine starting material enables facilitated access to perinatal tissues, which have extensive history of use in Western and Asian medicine. Such extracts are used for protective and immunomodulatory effects in various product categories. No therapeutic SPE preparations have been approved in Switzerland, yet unlicensed use has been documented in several private practices for mesotherapy (or as probable substitutes for original “living cell therapy”) [33,46].
Placental isotherapy	Formulation of patient-specific placental tissues into appropriate homeopathic preparations.	Placental isotherapy was commonly used until recently in Switzerland for various postpartum affections. Following medical prescription, thorough safety testing, and pharmaceutical magistral preparation, these products were dispensed to specific patients. Such preparations were notably available in Switzerland from Serolab SA.
Serocytol^®^	Equine immunobiologic products. Specific porcine tissues were transplanted to immunize horses, and the collected equine immunoglobulins were used to treat corresponding tissue-specific human affections.	The use of tissue-specific equine immunoglobulins was widely adopted in Switzerland since the 1930s, when Dr. Jean Thomas elaborated and democratized the practice of serocytotherapy. Specific porcine organs and tissues were transplanted in horses to generate immunoglobulins, which were then used as APIs in human medicine to treat affections of the corresponding organs and tissues. Several dozen pharmaceutical preparations (for oral, injectable, or rectal administration) based on this therapeutic principle were registered as therapeutic products in Switzerland by Serolab SA until 2020 [2,3].
Actovegin^®^	Deproteinized calf serum extract, in semisolid or liquid preparations.	Actovegin^®^ or equivalent products are highly used in injection form for circulatory affections and within professional athletic circles, for promotion of tissular repair and performance amelioration [47,48]. Actovegin^®^ is a registered therapeutic product, owned by the global Switzerland-based Takeda Pharmaceutical Company.
GM-1	Sialic-acid-containing glycosphingolipids, extracted and purified from mammalian nervous tissue.	Several neurotrophic and neuroprotective properties of GM-1 have been investigated, demonstrating potential roles and applications in neurodegenerative conditions. GM-1 has been produced by the global Switzerland-based TRB Chemedica SA. A similar preparation known under the appellation “Gricertine” was commercially available in Swiss pharmacies in the 1980s, that was presented as a central nervous system stimulant or protector, based on research around specific brain phospholipids [49].
Uro-Vaxom^®^ and Broncho-Vaxom^®^	Immunotherapy products containing complex bacterial cell lysates, formulated in dry oral form.	Such registered therapeutic products are used in the prevention of recurrent urinary or respiratory tract infections, respectively. They stimulate the immune system against potential pathogens [50,51]. These therapeutic products are registered and manufactured in Switzerland by OM Pharma SA.

**Table 2:** Comparative overview of selected and notable applicable legal and regulatory framework documents covering the development and practices of autologous and allogeneic cell therapy in Switzerland and in the European Union. High similarity existed in definitions, requirements, and possibilities between both considered and neighboring jurisdictions. It was noteworthy that in several instances, the European documents were applicable in part by extension to Switzerland. Most aspects concerning specific technical requirements for cellular therapies in Switzerland were derived from international (e.g., ISO, ICH), European (e.g., EMA, EDQM), and American (e.g., FDA) official sources. EC, European Commission; EDQM, European Directorate for the Quality of Medicines and Healthcare; EMA, European Medicines Agency; FDA, US Food and Drug Administration; ICH, International Council for Harmonization; ISO, International Organization for Standardization.

Legal/Regulatory Texts in Switzerland	Legal/Regulatory Texts in the European Union
Federal law on the transplantation of organs, tissues, and cells (Law on Transplantation, 2004)	Directive 2004/23/EC of the European Parliament and of the Council of 31 March 2004 on setting standards of quality and safety for the donation, procurement, testing, processing, preservation, storage, and distribution of human tissues and cells (2004)
Federal law on medication and medical devices (Law on Therapeutic Products, LPTh, 2000)	Directive 2001/83/EC of the European Parliament and of the Council of 6 November 2001 on the Community Code relating to medicinal products for human use (2001)
Federal ordinance on authorizations in the domain of therapeutic products (OAMéd, 2018)	Regulation (EC) No. 1394/2007 on Advanced Therapy Medicinal Products and amending Directive 2001/83/EC and Regulation (EC) No. 726/2004 (2007)

**Table 3:** Overview of notable public and nonprofit actors implicated in cell therapy development and clinical implementation in Switzerland, along with main cell therapy interests and identified industrial development partners. NA, not applicable.

Academic/Nonprofit Research Centers	Cell Therapy Interests	Industrial Partners
Lausanne University Hospital, Lausanne Burn Center	Skin (autologous and allogeneic solutions for burn wounds, donor site wounds, cutaneous ulcers)	ELANIX Sàrl
Lausanne University Hospital, Orthopedics and Traumatology Service	Cartilage (autologous chondrocyte implantation)	NA
University Hospital Basel, Department of Orthopedics and Traumatology	Cartilage (autologous chondrocyte implantation)	NA
Pediatric Burn Center, University Children’s Hospital Zurich	Skin (autologous solutions for burn wounds)	Wyss Zurich Regenerative Medicine Technologies Platform; CUTISS Ltd.
Swiss Stem Cell Foundation	Adipose stem cells (esthetics)	Technopark Zurich; Günter Leifheit Stem Cell Institute

**Table 4:** Overview of the various tissue-specific homologous applications currently considered or investigated at clinical and preclinical stages under the Swiss progenitor cell transplantation program using human primary progenitor cells. Described and respective stages of scientific and technical or industrial development are effective as of December 2021. API, active pharmaceutical ingredient; GMP, good manufacturing practice.

Tissue Type	Progenitor Cell Type Examples	Application Types	Considered Therapeutic Applications	Selected References
Skin	FE002-SK2 ^1^	Manufacturing: industrial GMP manufacturing transposition. Clinical trials: severe burns, refractory cutaneous ulcers, donor-site wounds.	Cutaneous wounds, burns, scars, grafting sites.	[9,19,23,26,70]
Cartilage	FE002-Cart ^2^	Manufacturing: industrial cell banking and product manufacturing. Preclinical studies: safety of transplantation in a caprine model.	Prevention of cartilage degeneration such as osteoarthritis. Treatment of critical cartilage lesions.	[44]
Tendon	FE002-Ten ^3^	Manufacturing: industrial cell banking and optimized API manufacturing. Preclinical studies: safety of transplantation in a lagomorph model.	Treatment of subcritical defects such as tears, or of volumetric tissue loss.	[45,82]
Bone	FE002-Bone	Manufacturing: optimized cell banking and manufacturing. Preclinical studies: safety of transplantation in murine and rat models.	Treatment of subcritical bone fissures. Treatment of critical bone lesions.	[79,80]
Muscle	FE002-Mu	Manufacturing: optimized cell banking and manufacturing. Preclinical studies: safety of transplantation in a murine model.	Treatment of subcritical defects such as tears, or of volumetric tissue loss.	[77]
Intervertebral disc	FE002-Disc	Manufacturing: optimized cell banking and manufacturing.	Treatment of critical intervertebral disc lesions.	Unpublished results
Lung	FE002-Lu	Manufacturing: optimized cell banking and manufacturing.	Prevention and/or treatment of inflammatory respiratory tract affections.	Unpublished results

^1^ The mechanically isolated cell type was deposited with the accession number ECACC 12070301-FE002-SK2 and with the reference number FIRDI BCRC 960460 in 2012. ^2^ The mechanically isolated cell type was deposited with the accession number ECACC 12070303-FE002-Cart and with the reference number FIRDI BCRC 960459 in 2012. ^3^ The mechanically isolated cell type was deposited with the accession number ECACC 12070302-FE002-Ten and with the reference number FIRDI BCRC 960461 in 2012 [83]. ECACC, European collection of authenticated cell cultures; FIRDI, Food Industry Research and Development Institute.

## Data Availability

The data presented in this work are available on request from the corresponding author. The data are not publicly available due to statutory and legal reasons.

## Abbreviations

ACI	Autologous chondrocyte implantation
API	Active pharmaceutical ingredient
ATMP	Advanced therapy medicinal product
CEA	Cultured epithelial autograft
CDEA	Cultured dermal–epidermal autograft
CHUV	Centre Hospitalier Universitaire Vaudois
CPC	Cell production center
DMEM	Dulbecco’s modified Eagle medium
ECACC	European collection of authenticated cell cultures
EC	European Commission
EDQM	European Directorate for the Quality of Medicines and Healthcare
EMA	European Medicines Agency
ePBB	Equine progenitor biological bandage
FBS	Fetal bovine serum
FDA	US Food and Drug Administration
FIRDI	Food Industry Research and Development Institute
GMP	Good manufacturing practice
HPL	Human platelet lysate
HUG	Hôpitaux Universitaires de Genève
ICH	International Council for Harmonisation
ISO	International Organization for Standardization
MCB	Master cell bank
PBB	Progenitor biological bandage
PCB	Parental cell bank
PMDA	Pharmaceuticals and Medical Devices Agency
QC	Quality control
SPE	Sheep placental extract
TFDA	Taiwan Food and Drug Administration
TrSt	Standardized transplant product
WCB	Working cell bank
